# Factors influencing clinician-educators’ assessment practice in varied Southern contexts: a health behaviour theory perspective

**DOI:** 10.1007/s10459-024-10341-3

**Published:** 2024-05-29

**Authors:** Danica Anne Sims, César Alberto Lucio-Ramirez, Francois J. Cilliers

**Affiliations:** 1 University of Oxford, Oxford, UK; 2 University of Johannesburg, Johannesburg, South Africa; 3 Technologico de Monterrey, Monterrey, Mexico; 4 University of Cape Town, Rondebosch, South Africa

**Keywords:** Assessment, Contextual factors, Faculty development, Global South, Health behaviour theory, Personal factors

## Abstract

In many contexts, responsibility for exit-level assessment design and implementation in undergraduate medical programmes lies with individuals who convene clinical clerkships. Their assessment practice has significant consequences for students’ learning and the patients and communities that graduates will serve. Interventions to enhance assessment must involve these assessors, yet little is known about factors influencing their assessment practice. The purpose of this study was to explore factors that influence assessment practice of clerkship convenors in three varied low-and-middle income contexts in the global South. Taking assessment practice as a behaviour, Health Behaviour Theory (HBT) was deployed as a theoretical framework to explore, describe and explain assessor behaviour. Thirty-one clinician-educators responsible for designing and implementing high-stakes clerkship assessment were interviewed in South Africa and Mexico. Interacting personal and contextual factors influencing clinician-educator assessment intention and action were identified. These included attitude, influenced by impact and response appraisal, and perceived self-efficacy; along with interpersonal, physical and organisational, and distal contextual factors. Personal competencies and conducive environments supported intention to action transition. While previous research has typically explored factors in isolation, the HBT framing enabled a systematic and coherent account of assessor behaviour. These findings add a particular contextual perspective to understanding assessment practice, yet also resonate with and extend existing work that predominantly emanates from high-income contexts in the global North. These findings provide a foundation for the planning of assessment change initiatives, such as targeted, multi-factorial faculty development.

## Introduction

In many contexts, final-year medical students are certified to graduate based on university-run examinations. In low-and-middle-income countries (LMICs) in the global South, designing and managing assessment can be the responsibility of the *individual* running clinical clerkships, referred to here as “clerkship convenors”. In contrast, high income countries (HICs) in the global North might implement (costly) programmatic assessment (van der Vleuten & Heeneman, 2016) and draw on the collective literacy of assessment committees. Critically, how assessment is designed has implications for student learning and public safety. Designing exit-level assessment in ways that yield both desirable learning effects and valid data for decision-making requires advanced assessment literacy. Ensuring sound assessment practice by *individuals* is therefore an important consideration for both curriculum leadership and regulators. The purpose of this article is to explore assessor behaviour, using Health Behaviour Theory (HBT) as a theoretical lens. In the process, the utility of HBT as a theoretical framework in this context is placed under scrutiny. Our research question was what factors influence the assessment practice of individual clerkship convenors in exit-level medical programmes in diverse Southern settings? In short, this is a qualitative study, situated in an interpretivist/constructivist paradigm, that sought to identify, and relate, factors influencing individuals’ assessment behaviours.

### Theoretical framework

Various factors impacting assessment practice, including its design and implementation, have been described in higher education and health professions education literature. However, these factors are described in a fragmented fashion rather than a systematic way that could enable an understanding of, and more strategic and effective interventions to enhance, assessment practice by assessors. Given our interest in enhancing assessment, if we conceptualise assessment practice as an educational behaviour by individuals, then Health Behaviour Theory (HBT) offers a useful theoretical perspective through which to consider how various factors may relate and influence practice (Cilliers et al., 2015, 2023). In other words, we are transposing HBT from its original *health* behaviour context to an *educational* behaviour context. In pursuing a rigorous application of the theoretical framework, we used it both to organised the literature review and for the secondary phase of deductive analysis, therefore a brief foray into the theory is in order.

**Fig. 1:**
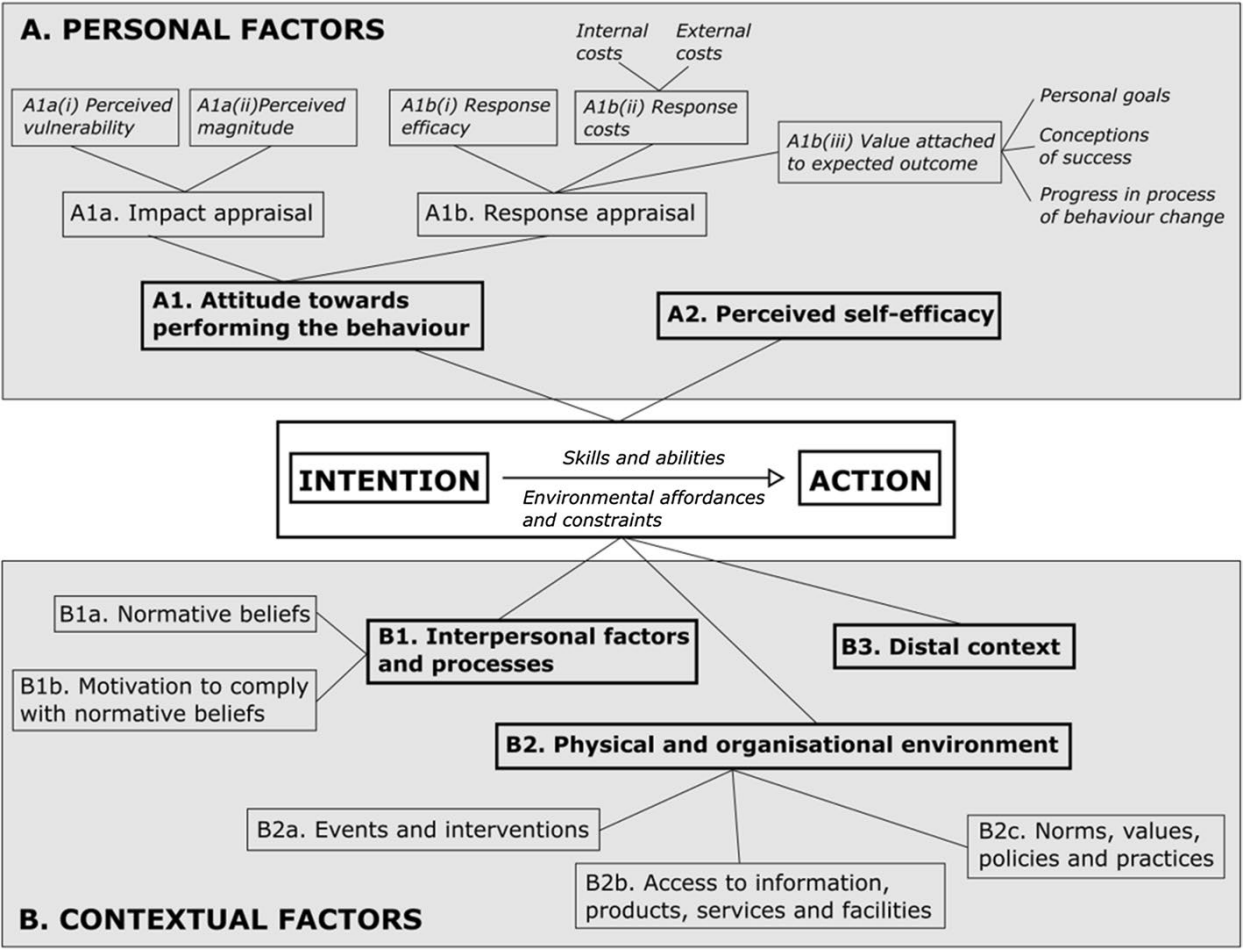
An eclectic model of Health Behaviour Theory (adapted from Cilliers et al. (2015)), as a theoretical framing for exploring the factors influencing assessment practice and change (see Table 1 for the expanded meaning of each construct in the context of assessment, and their positive and negative impacts). Personal factors that shape intention towards performing an action include attitudes and perceived self-efficacy towards performing a behaviour. Contextual factors that converge to influence intention to undertake a particular action include interpersonal factors and processes, the physical and organisational environment, and distal context. Relationships between factors are not simple; interactions may be multi-factorial and multi-directional, differing for different individuals. Intention formation is no guarantee that action will follow. The transition to action is itself mediated by the skills and abilities of an individual, and affordances or constraints within their environments

HBT is a family of theories that, broadly speaking, posit that behaviour results from complex interactions between personal and contextual factors (Glanz et al., 2015). HBTs offer distinct perspectives to variously explain and influence behaviour, each highlighting different factors as important elements in behaviour change. No single HBT can fully describe and explain a behaviour; rather in comparing HBTs, similarities and differences can be observed. For these reasons an eclectic model (Fig. 1) drawing on factors from different HBTs (Cilliers et al., 2015), that had previously been used to explore the learning effects of assessment (Cilliers et al., 2010, 2012), was used to inform this study (Table 1 provides explanations of each HBT element).

While the review will focus on individual constructs in the framework, it is important to note that these are interdependent and not mutually exclusive. The name and notation of each factor (Fig. 1, Table 1) are used in the text of the literature review and in the findings to signpost the reader in our alignment to the HBT model used.

**Table 1:** Examples and illustrative quotes (*italicised*) for each (A) personal and (B) contextual HBT element and their impact on (C) assessment intention formation and/or assessment action

HBT element	Positive influence	Negative influence
(A1) Attitude: How do I feel/what emotions do I experience towards assessment? (e.g., positive or negative)	I have a positive attitude towards assessment because… (see positives below, but, for example: assessment is seen as interesting, enjoyable, valuable and important) so I intend to/do practise assessment in a particular way Intention and action: T1: *“I was really fascinated with the idea about outcomes-based assessment … I like assessment; that’s why I was involved in it and I try to keep involved” (MX12)* See: Q1, Q4	I have a negative attitude towards assessment because… (see negatives below, but, for example: assessment is seen as a burden, nuisance or waste of time) so I do not intend to/do not practise assessment in a particular way Action: T2: *“Our main job is not doing assessment, it is running the ward and doing other things …* *It [assessment] is sometimes seen as a chore we have to do … It is … something you just have to get over with, so it is not always the most effort is put into it … [Assessment] is very much a little extra thing that we do, it is not our main focus” (SA13)*
(A1a) Impact appraisal: What effect will this assessment-related action have (or not)? *(A1ai) Perceived vulnerability*: How at risk am I, or others, to potential consequences of performing a particular assessment action (or not)? (e.g., likelihood of desired or adverse consequences) *(Alaii) Perceived magnitude*: How big are the consequences (or lack thereof) of a particular assessment action towards myself, or others? (e.g., size of desired or adverse consequences)	I intend to/do undertake a certain assessment action because this will likely yield positive, or avoid negative, personal consequences for me (e.g., gain favour with my boss; avoid getting in trouble with my institution); my students (e.g., achieve learning outcomes; pass the clerkship); their patients (e.g., treated appropriately; avoid complaints) Action: T3: *“The boss, the director, established [assessment practice] from the beginning, they say, ‘This is [how] this going to be’ … [we do it this way] because the boss says that” (MX8)* I do not practise assessment in a certain way because I do not think I could sleep at night knowing that there could be potentially unsafe graduates in the public healthcare system (magnitude), as I am responsible for assessment in my clinical block and ultimately patient care (vulnerability) *Intention and action: T4: “I’m in charge of the program, I have to look at these things. I do look at these things” (SA8)*	It does not matter if I do not intend to/do not practise assessment in a certain way because there is little risk/likelihood of any positive or negative personal consequences for me; my students; their patients (vulnerability); even if there are consequences, they will be limited (magnitude), therefore, I intend to/focus on other work (e.g., research, clinical) that the university is more interested in and will personally benefit me (e.g., institutional recognition and reward; career promotion) Action: T5: *“We’re all so busy with so many demands on us, that we can only set a certain amount of time for [assessment] … And then we are expected to deliver on research, which from our promotion point of view, in terms of how the staff thinks about it and the most important thing the university is interested in is the research output … So we’re working at the bare minimum in all these respects” (SA8)*
(A1b) Response appraisal: What is the likely outcome of undertaking a particular assessment action (or not)? *(A1bi) Response efficacy*: How effective is my response (or non-response) in achieving a particular outcome for myself, or others? (e.g., how aligned or valid a particular assessment action is perceived to be) *(A1bii) Response costs*: What price might I, or others, need to pay (or not) to do a particular assessment action? (e.g., the un/justifiable time, energy, resourcing, etc. costs required from myself, or others, to assess in a certain way) *(A1biii) Value attached to expected outcome*: How important to me (or not) is doing this assessment action? (e.g., undertaking a particular assessment action is perceived to be valuable, significant and important (or not) because it leads to success, achieves a desired goal, or contributes to a wanted change/progress)	This assessment achieves the desired outcomes (response efficacy) of competency development and safe future patient care (value), so I intend to/do it despite the costs to my personal time and energy (cost), such as implementing resource-heavy but valid and reliable programme of assessment (e.g., portfolios) Action: T6: *“I believe that irrespective of what obstacles get put in your way, we have an obligation to patients. So even if this is your curriculum and those are your core competencies, that is what they need to do. You cannot compromise and say, “Oh we need to cut out that number, we need to cut out that [from the in-course assessment].” That graduate is going out as an intern” (SA15)* Intention: T7: *“[The medical school] does not prepare students to pass an exam, it’s for them to be good practitioners and to be competent … Programmatic assessment is our goal” (MX6)* Action: T8: *“I understand the portfolio may be something more robust … At the end of the clinical rotation, the students have about 30 assessment forms of different aspects with different ratings … [The] portfolio gets everything; and I can have a better picture of my students” (MX12)* Education and assessment are personally important to me (value, success), which is why I intend to/do invest in them (e.g., earned an educational qualification, attend assessment training, put in effort despite little interpersonal assistance), to enhance my assessment practices and outcomes Intention and Action: T9: *“Especially in [names discipline], they have a big ego, and they know everything, and they know the world. When I started doing my Masters [in education], some people told me, ‘Why are you doing that? You’re wasting your time. No one is paying you more because you have a Master’s in education’ … I think it widened my … point of view and [I] try to get better at doing this [assessment] … I think it should be like encouraged” (MX10)* Action: T10: *“It was a massive workload for me, because… I didn’t get volunteers from the other people who participated in the module to assist in the marking, so I basically ended up marking everything myself… but I just sat one whole weekend and developed a urinary tract infection from sitting the whole time and marking… [But] it was easier for me to just sit one weekend and mark than waiting and running after people for marks” (SA11)*	This assessment does not add any value in achieving the clerkship outcomes (response efficacy), and it requires a lot of person-power (cost), so I intend to/will discontinue it because it is not worth it (response appraisal) Action: T11: *“We had to stop doing that [oral assessment] because of the burden of assessment and it was just taking up too much of the examiners’ time to be honest and we didn’t find that it added much value” (SA14)* My clinical work and patients are most important to me (value), and my clinical workload is great (cost), so I intend to/invest less time and energy into my assessment (e.g., using more convenient assessment methods like MCQs, rushing feedback to save time, or adjusting borderline marks to minimise additional workload such as re-assessing borderline students) See Action: T2 See Action: T5 Action: T12: *“MCQs are useful because they mark automatically and short answers are easier to mark … [they are more] convenient” (SA6)* Action:#T13: *“I fall into the mistake of, “Very good: 4, 4, 4, 4”, just like giving [students] all the points without really taking the time, not even if there is space to give like special feedback, I rarely use it, I don’t know, because of time … Time is probably the main problem” (MX5)* Intention: #T14*: “If the student fails the exam, it is quite an administrative nightmare … So, it is an incentive to not let them fail and if they are really bad, you’ll perhaps try and find a way to give them at least 50% … But having said that, if somebody is terrible, I don’t think we would do that. So, it is just for the borderline cases you might, because of the administration issue” (SA13)* Also: Q2–3
(A2) Self-efficacy: To what extent do I believe that I have the capacity (or not) to undertake a particular action? (e.g., self-belief in possession of personal abilities to undertake a particular assessment or assess in a certain way)	I feel confident in assessment design and implementation, so I intend to/will continue those assessment actions *See Action: T6*	I find assessment very challenging and lack confidence in my design and implementation abilities, so I do not intend to/do not undertake those assessment actions; instead I outsource my assessment practice/rely on others Action: T15: *“I think it’s [assessment] the most difficult things that I have to deal with … And, as I told you, I don’t have the answer. I don’t know. I’ve tried. We have jumped over models, different models, to see what it works … So, it’s been hard … It’s very difficult to make an assessment” (MX7)* Intention: T16: *“I can’t-, I don’t feel empowered to actually change things, because I don’t-, you know, that’s not my expertise … We actually rely on people [colleagues] with an educational background to help us develop assessment tools” (SA16)* Also: Q5
(B1) Interpersonal factors and processes: *(B1a) Normative beliefs*: What do others, whose opinions I value, think about assessment? (e.g., assessment beliefs by colleagues, students, patients, etc.) *(B1b) Motivation to comply with normative beliefs*: What peer pressure (or not) do I feel to practice assessment in a particular way? (e.g., the pressure felt to align with others’ versus personal assessment beliefs)	My colleagues believe that assessment should be practised in a certain way and they encourage me to do the same, so I intend to/do practice assessment in the same way Action: T17: *“[We do] what we are expected to do in the faculty, they tell you, ‘This is what you are supposed to do’” (SA15)* Also: Q6	Despite my colleagues having different assessment beliefs to me, I intend to/do practice the assessment that aligns with my assessment beliefs Intention: T18: *“[My assessment ideas] didn’t necessarily go through to the rest of the department … I don’t think there necessarily always an opportunity for my opinions … … [Because] if a guy says he’s a specialist in a [clinical] field, [then] there’s nothing you can teach him, ‘We’ve done it like that for years, so why would we change it?’” (SA10)*
(B2) Physical and organisational environment: *(B2a) Events and interventions*: What professional development events for assessment does my organisation offer (or not)? (e.g., assessment training, faculty development, in/formal educational opportunities) *(B2b) Access to information, products, services and facilities*: What assessment guidelines, support or resourcing (or not) does my organisation offer? (e.g., assessment support by a department or institution; available assessment rules and regulations of an institutional) *(B2c) Norms, values, policies and practices*: How important (or not) is assessment in this organisation and what are the prevalent ways the organisation practices it? (e.g., disciplinary, departmental or institutional culture of assessment)	My university offers assessment training which shapes how I intend to/do practice assessment (e.g., I have access to assessment training which influences how I think about, design and implement assessment) Intention: T19: *“The university does run various (assessment) workshops and things like that” (SA18)* There are university assessment rules and policies that provide guidance on assessment practice (e.g., I have access to institutional assessment rules and policies, which influences how I think about, design and implement assessment, such as aligning my assessment practices according to institutional regulations/I am confined or freed by university rules when wanting to innovate my assessment practice) Action: T20: *“The university’s rules stipulate [assessment practice] … We go about it the way we do purely because … the university recommends it” (SA1)* Also: Q7	My university does not offer any assessment training or support, meaning that I am left to my own devices when it comes to designing and implementing assessment (e.g., I maintain *status quo* assessment practices because I have no assessment training or access to new assessment innovation information to do otherwise) Intention: T21: *“[There is] no formal training programme for examiners unfortunately … In terms of examination techniques, we are all pretty much learning on the job” (SA16)* The culture of my university values and rewards research activities, for instance through career promotion, not educational or assessment actions, which discourages investment in assessment actions (e.g., I do not invest in my assessment practice because it is not valued or rewarded by my university) See Action: T5
(B3) Distal context: How does my broader context influence my assessment? (e.g., the social, historical, political, economic, national and international pressures felt—or not)	As an assessor who has experienced various inter/national socio-political movements (e.g., #RhodesMustFall student protests calling for free, decolonised education in South Africa) that highlighted the need for fair and non-discriminatory assessment practices, I intend to/do practice transparent and fair assessments that take student differences and cultural diversities into account Action: T22: *“I think assessment practices can always be improved, it is probably never perfect, but I think, by and large, the people do try hard to ensure that their assessment processes are as fair, transparent and accurate as what they can be. But I think the changes that have come about in the university and the emphasis that has been placed on, let’s call is decolonization, I think have brought those issues to the fore again … I think those challenges that the faculty have faced over the recent years has brought the issue of fair and transparent assessment to the fore, and I think that is as it should be” (SA18)*	As an assessor in a post-colonial context, which still values the colonial (Western, Euro-centric) gaze and strives to meet those international standards which are perceived to be better than local standards, I intend to/implement “international” assessment practices Intention: T23: *“I have to prepare you [the student], and you have to prepare yourselves, to take the exams, the international exams, not my exams, okay? … It’s very hard to get into residency here in this country [Mexico] … [We need to] take exams from abroad … at the international level and not my school [level] … I think that we have to move towards that direction… we have to move the assessment of every single class … [to] international standards, not ours, because we have to move up … I do believe that the main assessment has to be [at] international standards … I think we have to assess with international standards from day one” (MX4)*
(C1) Skills and abilities: Do I have the necessary competency required to undertake a particular assessment action myself (or not)?	I have the necessary personal competency to implement a particular assessment practice myself See Intention and Action: T9	I do not have the personal competency needed to implement a particular assessment practice myself See Intention: T15, Action: T16 Also: Q5
(C2) Environmental affordances and constraints: What factors in my environment facilitate or inhibit the undertaking a particular assessment action?	My environment has the necessary resourcing for me to undertake a particular assessment activity See Actions: T6, T10 Also: Q13	My environment does not have the resourcing needed for me to undertake a particular assessment activity See Actions: T2, T5, T13 Also: Q1, Q3, Q10-12

Quotes from the table are labelled “T”, in-text quotes “Q”

### Literature review

A. Personal factors

1. *Attitudes* towards assessment practice are shaped by appraising the *impact* (A1a) of adopting, adapting or performing an assessment practice (or not) and appraising the *response* (A1b) that will result from the assessment practice.
  Faculty who enjoy the challenge of innovating and improving assessment practices are more likely to change them (Bearman et al., 2016); whereas negative attitudes stifle change (Medland, 2018). Negative attitudes by faculty towards workplace-based assessment (WBA) discouraged their adoption (Massie & Ali, 2016). This particular example illustrates how the interaction of constructs shapes attitudes: a poor understanding of the purpose of WBA and a lack of belief that WBA would meaningfully achieve the desired outcome of developing student learning (A1bi) reflected in a low valuing of WBA (A1biii) (Massie & Ali, 2016). Perceptions of insufficient time for WBA in the context of higher clinical workloads (A1bii) also resulted in negative attitudes.

- *Appraising the impact* of performing any given assessment practice (or not) relates to perceptions of *vulnerability* (A1ai) to outcomes and the *magnitude* (A1aii) of those outcomes.
  Individuals in new positions of responsibility for assessment design in their courses (A1ai) felt pressured to redesign their assessments, or else risk potential negative consequences: not meeting student learning needs or performing badly in institutional reviews (A1aii) (Bearman et al., 2016). Similarly, individuals felt pressure to align assessments to professional standards or comply with regulators, at the risk of negative consequences: calling the quality assurance or accreditation of an institution into question (A1aii) (Medland, 2018; Skidmore et al., 2018).
- *Response appraisal* includes the perceived *efficacy* (A1bi) of an assessment practice in achieving a desired outcome; the internal and external *costs* (A1bii) associated with that practice; and the *value* (A1biii) attached to the outcome of the practice.
  Perceptions of *response efficacy* (A1bi) are influenced by faculty beliefs regarding the purpose of assessment. If the goal of assessment is seen as knowledge reproduction or application, faculty implement assessment methods that most effectively achieve those outcomes (Postareff et al., 2012; Sims & Cilliers, 2023a). If faculty believe that innovating assessment practice will *not* achieve their desired outcome, they are unlikely to change current practice (Halinen et al., 2013; Massie & Ali, 2016).
  Conversely, if the purpose of assessment is to support student development, faculty prioritise feedback (de Jonge et al., 2017; Halinen et al., 2013; Sims & Cilliers, 2023a). If faculty believe assessment should be objective, using portfolios for regular documentation and auditing are seen to be an effective response to achieve this outcome (de Jonge et al., 2017). However, if faculty are seeking to balance multiple purposes of assessment, they may respond with varied adaptations (Berendonk et al., 2013; de Jonge et al., 2017).
  Internal and external *response costs* (A1bii) positively and negatively influence how faculty respond towards assessment. Experiences of duelling professional identities and perceived conflicting roles (e.g., supportive educators versus judgemental assessor) discourage assessment action (Berendonk et al., 2013; Sims & Cilliers, 2023a). Resolution of professional identity (e.g., dual teacher-assessor) can reduce this perceived internal conflict (Berendonk et al., 2013).
  Clashing workload commitments (e.g., clinical versus educational responsibilities) exemplify high external costs (Karthikeyan et al., 2019). However, more experienced and qualified faculty were less affected by external costs (e.g., high student numbers, lack of time, quality assurance requirements) than novice faculty, and were better able to implement intended assessment (Norton et al., 2013).
  The *value* (A1biii) attached to the outcome of an assessment practice is impacted by personal goals and conceptions of success, both of which can relate to aforementioned professional identities of faculty. Where faculty found that their role as an assessor gave them voice in a conversation they valued, they felt successful as assessors because others listened to them, which encouraged their involvement in assessment (Medland, 2018). However, if faculty believed a researcher identity and practice constituted success (e.g., more recognised and rewarded), this discouraged investing time and effort into assessment (Bearman et al., 2016; Karthikeyan et al., 2019; Norton et al., 2019, 2013).

A2. *Perceived self-efficacy* is higher in more experienced faculty who feel more confident and capable in their assessment activities (Goos & Hughes, 2010; Norton et al., 2013), including item-writing (Karthikeyan et al., 2019), and decision-making (Berendonk et al., 2013).

B. Contextual factors

1. *Interpersonal factors and processes* include *normative beliefs* (B1a) and *motivation to comply* (B1b) with said beliefs.
  *Normative beliefs* (B1a), collectively held in departments and institutions, can hinder assessment redesign (Harrison et al., 2017; Roberts et al., 2021). Differing and diverse beliefs can influence the acceptability, use, effectiveness and therefore intention to practice certain assessment activities (de Jonge et al., 2017; Massie & Ali, 2016). In one instance, external examiners were selected based on whether or not they shared similar assessment beliefs with internal faculty (Medland, 2018).
  Interpersonal factors extend beyond peer interactions to relationships with students. The faculty-student relationship is central to the credibility afforded to feedback (Ramani et al., 2017). If students do not value particular assessment practices, that can impact on what faculty implement (Massie & Ali, 2016; Norton et al., 2019).
  For *motivation to comply* (B1b) faculty can be influenced by resistant or supportive colleagues (Harrison et al., 2017). Strong departmental leadership in assessment, in particular management of assessment resourcing, raising the ‘status’ of educational and assessment activities, and aligned personal beliefs around what good assessment is, can encourage shifts in assessment (Bearman et al., 2016). Yet, if colleagues viewed educational work, of which assessment is a part, as unimportant, this discouraged assessment innovation (Deneen & Boud, 2013) and existing practice was maintained (Bearman et al., 2016).
2. *Physical and organisational environment* (e.g., institutional and clinical) dimensions are: *events and interventions* (B2a); *access to information, products, services and facilities* (B2b); and *norms values, policies and practices* (B2c).
  *Events and interventions* (B2a) like institutional faculty development programmes can positively impact on faculty’s intention to implement desirable assessment practice (Bearman et al., 2016; Norton et al., 2013), and lack of assessment training opportunities can limit change (Karthikeyan et al., 2019; Medland, 2018).
  *Access to information, products, services and facilities* (B2b), such as assessment guidelines (e.g., for item writing) and quality assurance procedures, and committees to review assessments designs (e.g., blueprints) facilitate changed assessment practice (Karthikeyan et al., 2019).
  Assessment *policies and practices* (B2c) can constrain (e.g., institutional requirements promoting maintaining the status quo) (Bearman et al., 2016) or enable assessment innovation (Roberts et al., 2021).
  Regarding the culture of assessment, the *norms, values, and practices* (B2c) of disciplines, departments and institutions, have been reported to shape assessment practice (Bearman et al., 2016; Kogan et al., 2017; Medland, 2018; Ramani et al., 2017; Skidmore et al., 2018). For example, cultures may differ within residency programmes (Bing-You et al., 2019) and institution types (Norton et al., 2013, 2019).
  Institutional assessment cultures that stifle assessment change include a culture of fear, that seeks to maintain status quo and punish assessment innovation; and a culture of compliance, which emphasises meeting accreditation expectations (Skidmore et al., 2018). In contrast, a culture of student learning encourages learner-centredness in assessment (Skidmore et al., 2018).
  Faculty within a hierarchical institutional culture are discouraged from giving students feedback, whereas a familial culture encourages greater interaction between faculty and students, and thus feedback (Bing-You et al., 2019). Although, when an institution lacks clear assessment norms and has a vague or ‘nice’ feedback culture, the quality and impact of feedback given is limited (Ramani et al., 2017).
3. *Distal context* speaks to the broader social, cultural, economic and political influences that may shape assessment. While some studies describe the broader context of their research studies, few make explicit links to distal contextual factors as influences on assessment.
  National culture can impact on how faculty give feedback, how students receive it and what feedback is valued and effective (Ng et al., 2011; Suhoyo et al., 2014, 2017; Varela & Premeaux, 2008; Wilbur et al., 2017). For example, Indonesian students perceived feedback to be more instructive when it was jointly initiated by the supervisor and student and came from a specialist (i.e., a hierarchical national culture which values the authority of specialists) whereas Dutch students perceived feedback as more instructive when it was based on observation (i.e., a more egalitarian national culture) (Suhoyo et al., 2014, 2017).
  Studies from the global South (South Africa, Brazil and Indonesia) have shared how the complex national cultures, colonial histories, diverse and evolving socio-political and economic environments have tempered adoption of costly and contextually inappropriate assessment practices from the global North (e.g., OSCEs, standardised and simulated patients, national licensing examinations) (Burch & Seggie, 2008; Claramita et al., 2022; Mennin et al., 2022; Walubo et al., 2003). These are some reasons why established assessment practices are slow to change and other cost-effective assessment innovations are used instead (e.g., the use of more feasible structured orals, long case examinations, and portfolios; implementation of WBA with real rather than standardised or simulated patients for high stakes assessment; and language accommodations for diverse student populations) (Burch & Seggie, 2008; Claramita et al., 2022; Mennin et al., 2022).

C. Intention to action
  Intention formation is a precursor to action: the dynamic interaction between personal skills and abilities (C1), and an enabling or constraining environment (C2), influence transition of intention to action in pursuance of a specific goal (Gollwitzer, 1999). Intention is choosing and planning for assessment goals; action is the enactment and evaluation of said goals, plans, actions and achievements—or lack thereof (Achtziger & Gollwitzer, 2007)*.* Faculty may have positive intentions towards implementing or innovating assessment practice, yet be unable to convert those intentions into action due to lack of personal skills and abilities or factors in their environment that act as barriers (Bearman et al., 2016; Desveaux et al., 2021; Norton et al., 2019; Quesada-Serra et al., 2014).

1. *Skills and abilities* of faculty mediate intention to action: the more experienced, trained and assessment literate faculty are the more likely they are able to enact or change their assessment practices (Berendonk et al., 2013).
  Interestingly, others have found that some faculty de-emphasise personal competencies as a mediator and stress the larger system or environment of assessment as a greater driver of assessment enactment (Norton et al., 2013, 2019).
2. *Environmental affordances and constraints* relate to broader external factors that may enable or hinder the intention to action transition. For example, poor assessment practice (e.g., low quality item writing) cannot be solely attributed to a lack of personal skill by assessors; rather, other structural-level factors need to be taken into account. These include lacks of institutional support in terms of governance, training, importance allocated to educational activities, time dedicated to educational activities, assessment costs and logistics (Karthikeyan et al., 2019).
  Many studies report time, personnel, finances, infrastructure and workloads, as constraints on potential assessment actions (Bearman et al., 2016; Deneen & Boud, 2013; Goos & Hughes, 2010; Massie & Ali, 2016; Norton et al., 2013). Faculty may intend to use assessment for learning yet due to institutional assessment rules they are limited in the assessment actions they can perform (Bearman et al., 2016; Deneen & Boud, 2013; Goos & Hughes, 2010; Norton et al., 2019; Quesada-Serra et al., 2014). External examiners too may intend to provide specific and critical feedback, yet due to an environment unwilling to change, alter their assessment behaviour and give ineffective feedback in the form of broad and generic comments (Medland, 2018).
  In summary, while various personal and contextual factors have been identified to influence assessment, these emanate from diverse sources in terms of time and place, with inconsistent description in the sources cited of the distal context in which the findings were made. Reports typically do not offer systematic and coherent understandings of how various factors interact to influence how and why faculty practice assessment in the ways in which they do. Given the evident impact of distal context on practice, it is notable that the vast majority of the literature cited draws on the experiences of assessors and assessment in the global North.
  Difference in how assessment is practiced should be further considered within a broader understanding of contextual difference. Assessment is a socially-situated and interpretive practice, impacted by those who design and execute it, who, in turn, are influenced by their contexts (Hanesworth et al., 2018; Shay, 2004). We (Sims & Cilliers, 2023a; Sims, 2023b), and others (Haug et al., 2021; Kloß, 2017; Sud & Sánchez‐Ancochea, 2022), understand that the terms ‘global South’ and ‘global North’, or the North–South binary, can be problematic; reinforcing the dichotomy and homogenising heterogeneous contexts. However, being able to broadly compare collectively different settings, not just economically, as the terms HIC and LIMC emphasise, but socially, culturally, politically and historically, in contrast to the assumed norm, is important—especially as these ‘other’ contexts do not reflect those that dominate medical education literature (Maggio et al., 2022; Naidu, 2023). We have previously documented some of these historical, economic, educational and health differences (Sims & Cilliers, 2023a). For these reasons we use global South and global North to both highlight the broad multi-factorial differences between these meta-contexts and to resist global power and knowledge hierarchies. In short, the epistemic and pragmatic differences between the assumed hegemony of the global North and the global South cannot be ignored (Connell, 2013; Naidu, 2021a; Paton et al., 2020; Sims, 2023b). Acknowledging the impact of context, our research question was what factors influence the assessment practice of clerkship convenors in varied Southern contexts.

## Methodology

Given the pivotal role of clerkship convenors in assessment in their contexts, participants were clerkship convenors from varied Southern settings. Data was collected through in-depth semi-structured qualitative interviews. Convenience sampling was employed at two public (state) research-intensive universities in South Africa (eighteen participants; labelled “SA”) and one private non-profit teaching-intensive university in Mexico (thirteen participants; labelled “MX”). Authors Sims and Cilliers organised the interviews in South Africa, Lucio-Ramirez in Mexico. Despite the use of convenience sampling, there was participant diversity in terms of gender, career stage (early, mid and late), academic position (senior lecturer through to full professor), clinical disciplines (bioethics, community health, emergency medicine, family medicine, haematological pathology, paediatrics, public health, medical virology, neonatology, neurology, obstetrics and gynaecology, internal medicine, surgery) and contexts (colonial histories seen in the languages of instruction, and institutional and national cultures) (see Sims & Cilliers, 2023a, for detail on educational and sociocultural contexts).

Ethical clearance from participating universities was obtained. Participants were invited to participate via email including a brief explanation of the study and purpose of the interview. Thirty-one (31) clerkship convenors were interviewed: 12/12 from the first South African university, 6/8 at the second South African university, and 13/60 from the Mexican university (31/80 = 38.75%). Once-off, one-on-one, in-person interviews took place at a time and location of the participant’s choosing (clinical and university settings). Informed consent was obtained after introducing the study topic at the start of the interview but before any questions were posed. Reasons for nonparticipation were not explored as there was no follow-up with nonresponsive invitees.

Cilliers and Sims conducted the interviews in a language of participants choosing (i.e., Afrikaans, English or Spanish). Reflexive preparation (Sims, 2023a) for data collection took place through a pilot interview, for trialling of the interview question guide and rapport development, through background reading on sampling contexts and conversations with local insiders. The pilot did not contribute to the study dataset. At the time of data collection, Sims was a young, White female, (non-health professional) doctoral student and novice qualitative researcher (from a basic sciences background) with no relationship to any of the participants. Cilliers and Lucio-Ramirez, both older males, of White and Latino backgrounds respectively, were senior medical professionals and educationalists (i.e., professors and employed academics at the universities sampled), and colleagues of the participants who drew on existing relationships for recruitment. All authors were citizens of the countries sampled (Sims and Cilliers South Africa and Lucio-Ramirez Mexican) and had undertaken educational training in these contexts. In drawing on critical perspectives required for meaningful reflexivity, it was important for research done in varied Southern contexts to be undertake by Southern researchers working with Southern peoples, who were not just familiar but embedded within these settings, understanding the socio-historical and cultural realities and nuances of these context that could affect participant assessment thinking and practice, and interview power dynamics (i.e., local and insider perspectives). Moreover, Cilliers had previously used HBT as a theoretical framework for assessment research exploring student learning behaviours (Cilliers et al., 2010, 2012). Shared lived experience, and relevant practical and theoretical expertise, informed data collection and analysis.

Interviews were conducted with the broad goal of identifying not only *what* factors might influence assessment practice and but *how* and *why*. Care was taken to explore behind the manifestations of practice that were surfaced during interviews, loosely guided by the sensitizing concepts of personal and contextual influences from the guiding theory. As HBT provided a theoretical framework for the study, an opening neutral prompt of, “How do you practice assessment in your clerkship?” was used to put participants at ease and enable accessible dialogue. Then, the following interview questions were asked: “What personal factors influence your assessment practice?” and, “What contextual factors influence your assessment practice?” The specific HBT factors (Fig. 1) were not used as probes during the interviews but inferred during analysis. Participants’ responses dynamically guided future interviews, with more specific prompts integrated in light of ongoing and recursive data collection and analysis.

Interviews were audio-recorded and transcribed verbatim. Field notes were taken during each interview which were consulted during transcription, coding and analysis. Prompt transcription and coding, with repeated readings of the transcripts, enabled familiarisation with the data. Initial coding was first undertaken inductively using NVivo by Sims, followed by deductive analysis that was theoretically-sensitized using the factors of Cilliers et al. (2015) HBT model. While HBT offered a helpful lens for understanding assessment practice, we were careful to identify and discuss seemingly incongruent findings. These could ultimately all be related to factors in the model.

Accordingly, coding, searching, reviewing and defining of themes took place iteratively, throughout the interview process and over multiple rounds of critical and reflexive discussions by Sims and Cilliers. An independent reviewer, an educationalist colleague, randomly selected transcripts for coding and comparison to the emerging findings. Moreover, independent review of our proposed findings by Lucio-Ramirez took place once data collection, analysis and results writing were ‘complete’. It was important to have authors from both contexts (South Africa and Mexico) to review the trustworthiness (i.e., ecological validity and credibility) of the proposed findings.

The concept of data adequacy, informed by information power, conceptual depth and theoretical sufficiency, was used to justify the sufficiency of our sampling and the cessation of data collection and analysis (Sims & Cilliers, 2023b). Dialogue was relevant and strong, given that participants were experienced in convening assessment. Interviews were rich and deep, each lasting 45–60 minutes. Extensive evidence from the data is presented here to rigorously support claims made, with strong resonance to theory.

### Findings

Personal and contextual factors shaping assessment practice were identified. In some instances, there were clear links between factors and practice; in others, there was evidence of intention formation which may, or may not, have resulted in practice. Importantly, while individual factors are discussed below, the relationships between each HBT factor and assessment intention/action were not necessarily linear or one-to-one; rather, they interacted with a number of other factors in a complex interplay. Therefore, quotations usually contain a number of interacting factors are referred to throughout. Table 1 provides an explanation of the meaning of HBT constructs in an assessment context and illustrates the relationships—each of which can be positive or negative—between the factors and assessment practice. The table serves as an overview of the findings and as an aid to link the theoretical framework (Fig. 1) with the more detailed text elucidating the role of each factor.

A. Personal factors
  Attitudes (A1) influenced assessment behaviours negatively and positively (T1). Negative attitudes discouraged assessment practice; investing time on assessment was not prioritized (Q1, T2, T11):
  Q1: “In practice the demands on our teaching, especially … the undergraduate load and what we are trying to do … it is not feasible … There is so much pressure, and that pressure is increasing to do all kinds of other things as well; research, getting involved with the province [state], attend meetings, go to courses that the university thinks are good for us, for the students … and then trying to keep up with professional … reading and interaction … Assessment, quite frankly, if I were to summarise it in one word, a nuisance” (SA12).
  When appraising the impact (A1a) of undertaking a particular assessment behaviour (or not), convenors considered the likelihood and the magnitude of the impact (A1aii) on themselves, students and patients. There was a risk of negative personal consequences for not complying with mandated assessment practices (T4). Yet, if there are not only no consequences for poor assessment, but also no reward for quality assessment practices relative to other activities for career progression, then impact appraisal had a doubly negative influence (T5).
  As convenors, participants felt responsible (A1ai) for the clerkship assessment practices (T4). If they believed that their assessments significantly impacted on student competency development and future clinical practice, they practiced assessment that addressed competency development required for graduates’ future work as junior doctors—even if there were significant costs (A1bii) (T6, T10).
  Response appraisal that a particular assessment action was not efficacious (A1bi) in achieving intended assessment purpose resulted in cessation of that action (e.g., orals; T11). This was compounded by high assessment costs (A1bii) (e.g., time, labour) and if clinical and research work was more valued (Albiii) than educational and assessment activities (T2). Assessment practices (e.g., MCQs; Q2, T12) that freed up time for other more valued pursuits were adopted (Q5).
  Q2: “You want something that’s easy to mark, quite frankly, that’s probably the most [important] ... you sit with 180 exam papers … it’s a weekend in your life that’s just gone” (SA12).
  Even if a convenor believed that their assessment achieved beneficial effects (A1bi) and significant impact (A1aii), burdensome workload (A1bii) could adversely affect assessment practice e.g., rushing through feedback (Q3, T13) or feeling pressure to adjust borderline student grades so as to minimise additional administrative burden (T14).
  Q3: “Sometimes they [colleagues] don’t pay attention. That’s one of the most important problems … [They] just put, “Excellent, excellent, excellent, excellent” … We have really busy schedules and really busy clinical rotations … we have been getting more students and bigger groups and we are not doing that well” (MX12).
  Convenors who believed that future patients were vulnerable (A1ai) to the beneficial effects of their assessment practices (A1bi) were not prepared to compromise assessment design (T6). Therefore, despite high personal cost (A1bii), they used assessment methods better suited to decisions about clinical competence (T6–8). Moreover, some pursued educational training and qualification rooted in their desire to be effective in their educational and assessment duties (A1biii) (T9). This reflected in more positive attitudes towards assessment as necessary and important, supporting assessment practice (Q4, T7–8).
  Q4: “Each [assessment tool] interrogate[s] different things… they all have their strengths and their weaknesses … We want to ensure that they’ve covered the theory, so, and they’ve read the text and what we want them to know; so that’s the point of assessment one. The point of assessment two is… to test their application ability; can they apply the knowledge to clinical scenarios… Assessment three, the oral and portfolio, make sure that we interrogate something that they’ve done during the block… and that they’ve read around the cases… The fourth exam interrogates actual practical clinical… skills… So, each of the formats does sort of assess different things” (SA18).
  A low sense of self-efficacy (A2) towards assessment discouraged intention formation and action, leading convenors to rely on other more educationally-experienced colleagues for assessment design (Q5, T15–16).
  Q5: “I’m a clinician, I am not [a teacher], I haven’t got any experience in education and my main interest is clinical work… So, a lot of our direction comes from [a colleague] because they’ve got postgrad experience and training in education … They actually designed our portfolio mark sheet [rubric]” (SA14).
B. Contextual factors.
  Contextual factors influencing assessment practice include interpersonal, physical and organisational, and distal factors.
  A strong assessment leader could inspire, through role-modelling and personal support (Q6), or force (T3), assessment change (B1).
  Q6: “There is sometimes a struggle with resistance, [but], plenty of the faculty believe in me, trust me and they also respect me as a leader … I go into [assessment] business personally … I personally support people. For example, yesterday I was helping someone with [their] assessment at 10pm” (MX6).
  On the other hand, encounters with unsupportive colleagues (Q3, T9-10), rooted in differing professional beliefs and goals (B1a) (e.g., valuing clinical work more than educational duties), dampened convenors’ assessment re/design intention. High motivation to comply with normative beliefs (B1b) reinforced existing practice (T18).
  The physical and organisational environment (B2) impacted on assessment through the provision of assessment events and interventions (B2a); for instance the availability (T19) of assessment training (or not) (T21).
  Access to assessment-related information, such as university assessment rules (B2c), influence how assessments are practiced. A convenor may want to adapt an assessment, yet be stifled by the assessment regulations of their university (T20), whereas others have the freedom to innovate without restrictions (Q7).
  Q7: “The institution is pretty flexible … the institution gave us a lot of freedom so we can perform what we think is best for our students” (MX8).
  The availability of educational and assessment expertise (B2b) influenced assessment design (Q5, T16).
  Departmental and institutional values (B2c) motivated or restricted (T17) assessment and the degree of investment in assessment (T2, T5).
  The influence of social, historic, economic and political pressures of the distal context (B3) diverged when comparing the South African and Mexican contexts of clerkship convenors. Moreover, recent calls for decolonizing higher education in SA had emphasised the need for assessment of diverse student populations to be fair and equitable (T22).
  A South African participant, within the ‘post’-colonial and ‘post’-Apartheid context of SA, perceived pressure to pass, to maintain throughput of, historically excluded  students despite personal assessment beliefs, as pass rates were linked to public institutions receiving funding subsidies from the government:
  Q8: “We’re all affected by politics. Politics is really about decisions to how the money goes … [The] cry, “Pass one, pass all” from a political point of view is obviously completely misguided and misunderstanding what qualifications are for. But it is also something that the [state] encourages, because, as in many of the weaker [historically racially-segregated under the Apartheid government] institutions, you’ve got financial problems; if they fail a significant proportion of students, they [institutions] are financially embarrassed. So that is a perverse incentive and it makes it very difficult for the previously disadvantaged university … I think that there needs to be a much more subtle, complex, way of funding and providing money for … disadvantaged universities” (SA12).
  In SA, expansion of higher education to include previously excluded and disadvantaged students, also affected convenors. Convenors perceived pressure to pass students (Q9) due to the pragmatic challenge of a growing student population.
  Q9: “In the past many [previously excluded students] had a language problem [i.e., English was not their mother tongue], but that has been sorted now. And if they did really poorly we found that some of us adjusted their marks so that it could reach a pass … The political pressure wasn’t just in terms of colour [ethnicity], but based on, you can almost say, a university political pressure. You can’t fail twenty students. Only ten are allowed to fail or no more than five are allowed to fail. And it’s not about colour or the language of the students in this case. It’s about the volume … What do you do when you suddenly have minus twenty or plus twenty in the new year? That political pressure is greater than the socio-political pressures of colour and language” (SA7).
  For Mexican participants, national culture that aligns strongly with maintaining tradition negatively influenced assessment in the form of resistance to changing practice. Yet, strong assessment leadership (T3, Q6) or institutional rules (T20) could force assessment action in particular directions despite resistance.
  Mexican convenors additionally spoke of the pressure of their institutional culture to continually evolve, and more specifically “internationalise”, their assessment practices, implementing dominant assessment trends, e.g., programmatic assessment (T7). One reason for this pressure was the limited capacity for residency training in Mexico, along with a valuing of specialising and sub-specialising over (poorly paid) general practice. Convenors wanted to shape their assessment to enable graduates’ residency and specialist training in Mexico and beyond (T23). This is in contrast to the strong primary healthcare philosophy and practice in SA (Hartman et al., 2012), in which general practitioners operating at a primary level are needed and valued.
  This pressure was confounded by high tuition fees at the Mexican institution and resultant expectations from students and their parents that this educational training would guarantee a bright future (Q10).
  Q10: “Our students, they pay a lot to get into our institution, their dream is to get into residency [programmes] ... As the institution, we have to take that in mind and it’s our responsibility” (MX4).
C. Mediators of assessment practice.
  Conversion of intention into action appeared to be mediated by personal assessment competency (i.e., assessment-related knowledge and skills/abilities of clerkship convenors), and environmental affordances and constraints (e.g., availability of resources).
  If faculty lacked the necessary competencies (C1) they did not take ownership or enact assessment themselves, but outsourced design and implementation to other colleagues (Q5, T16). However, faculty who had undertaken educational training described enhanced assessment competencies and practices, attached greater value to assessment and offered resistance to constraining normative collegial beliefs and disciplinary cultures (T9).
  There were numerous environmental barriers (C2) hampering assessment practice and change. Despite positive intentions, assessment practice was constrained by feasibility issues; high workload demand from large class sizes and high clinical workloads, confounded by a lack of human (e.g., patients, assessors) and financial resources (see Q1, Q3, Q11-12, T2, T5, T13).
  Q11: “There are a lot of students and due to the lack of manpower we sometimes have to do [assessment] in a very short manner. And we have to know that we can’t cover the whole field… We try to identify what we feel is more important or appears more frequently, then some things are unfortunately neglected … When you have to assess a student, you would like to take a whole day and sign him off [as competent] … But you only have half an hour or two hours to assess the whole year, I think most people would agree with me, that it is actually impossible.” (SA7).
  Q12: “We use the patients that are available… if those are difficult or easy cases, then the student’s performances are also going to be dictated by what they see … I told you about the resource constraints... An adequate assessment would be adequate patient material and an adequate number of staff – we don’t have that … So, in my mind it’s not a perfect exam, but it’s a satisfactory exam, given the constraints” (SA8).
  All convenors were jointly employed by both the university and state in educational and clinical capacities respectively. Some also undertook private clinical practice (dictated by familial financial needs and personal conceptions of success; Q13). The resultant workload constrained intention enactment. Even if institutions provided increased access to assessment-related information, products, services and facilities, such as training workshops, environmental constraints limited faculty engagement in them.
  Q13: “I have to do both [clinical and educational work] in order to get money to survive ... So, it consumes me, [it takes] a lot of time doing both. I also have to do something for the HEI [university], which [also] consumes me [and takes] a lot of time. When you are doing these three things [clinical, research and educational work], and they ask you to go to a [educational] course that lasts three hours, usually nobody wants to be there and [they] don’t pay attention at all, answering phone calls from the hospital, from patients, we are not that really into the course … [Clinicians] have to cancel their practice in order to go to those [training] programmes. And we don’t get paid by going to these programmes. So, they lose money, and they complain about that” (MX2).
  However, other convenors, based on the strength of their personal beliefs and convictions around assessment and education more broadly (A1a, A1b) enacted planned assessment e.g., giving quality feedback for student learning, despite workload demands (Q14).
  Q14: “What is your responsibility as a lecturer? Is it my responsibility to do that [give feedback]? Am I getting paid to do that? And then I say, “No, you are not getting paid.” It is just part of your view on life. I want to help them [students] as far as possible, but with all the other responsibilities you have, it makes it a little difficult at times. Time is a factor… I have to decide whether I am going to invite the students to come talk with me in my office for an hour, or am I going to use that hour to mark a few question papers? Then I say, “I definitely don’t have to invite them to come and talk to me. I probably don’t have to do it. Nobody is going to blame me if I don’t do it”, but I know if I decide to do it, it is going to mean something to them” (SA7).

**Fig. 2:**
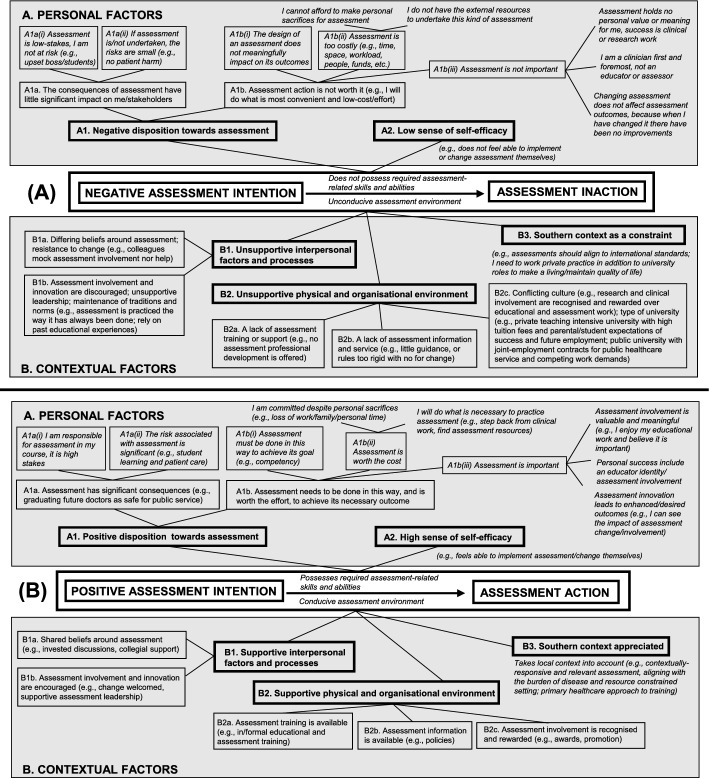
An overview of the interaction between personal and contextual influences on assessment intention formation and action in varied Southern contexts in South Africa and Mexico. Any given factor can influence assessment intention formation positively or negatively. In this study, we found more negative than positive influences on assessment practice. For any given person, some factors can have a positive influence, others a negative influence. For the purposes of illustration, collated negative (part A), and positive influences (part B) are illustrated here

## Discussion

This study explored factors influencing assessment design and practice by clerkship convenors in varied Southern contexts (Fig. 2). HBT provided a useful lens to relate interacting personal and contextual factors contributing to assessment intention-formation and action. The effects of these factors were found to shape *how* and *why* assessment is implemented in the ways that is it. While previous research typically explored influencing factors in isolation, this study used HBT to systematically and coherently describe and explain assessment practice. It further contributes to research demonstrating the utility of HBT to describe and explain education practice (Cilliers et al., 2015, 2023).

Using HBT to interrogate existing literature when framing this study now also allows for a theoretical consideration of how these findings relate to other work. For instance, Massie and Ali (2016) describe negative attitudes towards WBAs, flowing from a lack understanding of the purpose of this assessment methodology, insufficient time and inadequate training of assessors. HBT would relate these to response efficacy (A1ai), response costs (A1aii), available interventions (B2a) and access to information (B2b). Furthermore, our model suggests additional factors that could influence a particular assessment practice, such as differing professional identities (clinician identity prioritised), value systems (clinical workload prioritised) (A1biii) and assessment-related beliefs (not relating to student learning or future patient care) (A1ai, A1bi). Additionally, a lack of supportive colleagues and strong assessment leadership (B1), and a broader culture of assessment that does not value or reward assessment activities, but rather research (B2c) could shape these negative assessment intentions and actions. In other words, observations of ‘negative attitudes’ towards assessment by clinician-educators could merely be an observable ‘symptom’, with the underlying ‘pathophysiology’ elucidated by HBT.

Another factor influencing assessment practice is conceptions of assessment (Sims & Cilliers, 2023a). Various components of conceptions map to factors identified here. For instance, assessor beliefs around the purposes of assessment, their temporal impacts and whom their assessment is accountable towards; their roles and responsibilities as assessors; and emotional valence relate to response efficacy (A1bi), perceived magnitude (A1aii), perceived vulnerability (A1ai), and attitude (A1) respectively. The associated assessor characteristics of assessment literacy, professional identity and self-efficacy relate to personal skills and abilities (C1), values (A1biii) and perceived self-efficacy (A2) respectively. The relationship between the factors explored here and conceptions of assessment remain to be explored in greater depth.

The impact of distal contextual factors has not been extensively described, and the majority of literature originates from the North, which requires translation for transferability by practitioners and researchers in the South. Our findings add a particular contextual perspective to understanding assessment practice, while resonating with and extending existing work that is predominantly emanates from the global North. For instance, workload pressures experienced by convenors employed jointly by clinical and educational institutions (A1bii; C2) appear rooted in socio-historical (i.e., colonial) realities, such as high student to clinician-educator ratios, and heavy clinical workloads in areas with higher burdens of disease and low healthcare resourcing (Sims & Cilliers, 2023a).

It has been suggested that the limited success in effectively changing educational practice in lasting ways results from not using educational research and theory to inform change initiatives (Albert & Reeves, 2010; Bordage, 2009; Gibbs et al., 2011; Onyura et al., 2017; van der Vleuten & Driessen, 2014). This study presents a holistic theoretically—and empirically-grounded framework explaining high-stakes, exit-level clerkship assessment practices designed and run by convenors. It illustrates how assessor behaviour is shaped positively and negatively by an array of factors. The factors described in our model offer compelling targets for faculty development and assessment enhancement, mitigating negative factors (Fig. 2, part A) and upholding positive factors (Fig. 2, part B), while reinforcing that a multi-factorial approach is needed for effective outcomes (Onyura et al., 2017; Sorinola et al., 2017).

In terms of targeting personal factors, interventions should develop assessor beliefs (A1a, A1b), including taking ownership of their assessment as responsible convenors (A1ai), along with appropriate competency required to design and implement clerkship assessment themselves (A2, C1). Additionally, interventions at the contextual level (B) should address *all* the factors mentioned; not just strong assessment leadership and apprenticeship (B1) but also increasing access to assessment-related information (B2c, e.g., policy documents, that encourage quality evidence-based assessment practices) and interventions (B2a, e.g. training programmes). Institutions should review and adjust workload models to enable attendance of jointly-employed faculty (e.g., protected time for educational training and activities) (Onyura et al., 2017). Moreover, they could reward attendance of such training (e.g., continued professional development points, an accredited certificate, access to additional training and funding opportunities, recognition in promotion, etc.). The culture (B2c) of assessment too could be disrupted through increasing recognition of quality assessment practice (e.g. institutional awards for assessment innovation). Assessment review processes should be put into place, whereby quality assessment practice is recognised and rewarded (A1biii). Requiring the attendance of entire departments as assessment training, with meaningful consequences for nonattendance (Ala), could help to challenge normative beliefs (B1a) and assessment cultures (B2c)—yet care must be taken not to adopt a punitive approach that can discourage change implementation (Cilliers & Herman, 2010). The broader culture of disciplines, departments and institutions should be considered when designing faculty development; for example, increasing collaboration across diverse stakeholders to encourage changed beliefs, values and practices (Lewis & Steinert, 2020). In reconceptualising faculty development from individual-level interventions, clinician-educators involved in assessment should be collaboratively developed within teams (i.e., departments and organisations) in authentic environments for more sustained change (Cilliers & Tekian, 2016; Morris & Swanwick, 2018; Onyura et al., 2017).

Critically, institutions should ensure that convenors have a conducive environment with the necessary resourcing (e.g., assessment staff, clinical material, assessment question bank, venues, etc.) needed for their assessment practices (C2), or else, despite desirable assessment intentions developed through faculty development, convenors may be unable to enact them—as our findings show. This raises a particular challenge for environmentally-constrained settings in the global South; without systemic increase of healthcare and education resourcing (human, financial and infrastructural), the pressures of disease burden, patient numbers and a lack of time for educational work persist. While these realities are very difficult to change, they provide opportunities for creative and pragmatic, but valid, assessment solutions (Burch & Seggie, 2008; Claramita et al., 2022; Mennin et al., 2022; Walubo et al., 2003).

In a similar vein, as the field of Health Professions Education comes to reckon with its colonial roots (Naidu, 2021a, 2021b, 2023; Paton et al., 2020; Wyatt, 2022), the contextual-appropriateness of assessment practices and interventions has been highlighted. The desire for internationalisation of assessments expressed by some, while partly informed by limited residency availability (Santana-Davila & Martinez, 2004), could also be argued to be ‘colonial’ (Rashid & Griffin, 2023). ‘Best’ practice recommendations from the global North are not inherently universally applicable, nor feasible in settings of the global South (Rashid & Griffin, 2023). A critical stance in evaluations of ‘good’ assessment and being cognisant of context and culture should inform practice.

In summary then, these findings highlight how new interventions limited to faculty development only are likely to yield limited results. In resource-constrained environments, there are also limits to what can be implemented; therefore contextual-awareness is needed. The exception here might be where faculty have a strong sense of self-efficacy and more developed conceptions of assessment (e.g., ‘active owner’) (Sims & Cilliers, 2023a). These recommendations of multi-factorial (personal, contextual) and multi-level (individual, interpersonal, organisational, environmental levels) intervention are aligned to faculty development and transfer of training literature (Cilliers & Tekian, 2016; Onyura et al., 2017).

## Limitations and future directions

While HBT offers rich and complex perspectives on behaviours, it is only able to account for a proportion of behavioural variance (Cilliers et al., 2015). However, it was chosen for its systematic representation of behaviour, including its interactions between personal and contextual factors, in comparison to isolated descriptions of factors in the literature.

Although HBT was broadly used to sensitise the development of the interview guide, participants were not asked about specific personal and contextual elements; rather, the broad and open-ended questions sought to avoid potential HBT confirmation bias. Nor did we specifically ask participants to distinguish between their assessment-related intentions and actions. Rather, alignment of participant responses to each HBT factor, intention-formation and action, were inferred in participants’ responses. While many of the quotes infer intention, and indicate the possibility of transition to action, intention-alone can be a poor predictor of action (Achtziger & Gollwitzer, 2007; Gollwitzer, 1999). Additional primary data, such as assessment observations, and secondary data, such as assessment artefacts (as opposed to self-reported interview data alone), could provide further evidence of links between factors and practice—or lack thereof.

Given the use of convenience sampling it is likely that participants were more interested in assessment practice than those who opted not to participate. This makes some of our findings of even greater concern, as factors constraining assessment practice may be more influential in the latter group.

While a strength of this study is its multi-site sampling across varied Southern contexts, these findings and implications may not be transferable to other settings, despite general alignment between the factors identified in the (predominantly Northern) literature review and the proposed model of assessor behaviour and its influences. As aforementioned, the global South is not a monolith but diverse. While sampling is not representative of the global South as a whole, this study contributes evidence from underrepresented Southern contexts.

Sampling was specifically limited to clerkship convenors given the longer-term goal of targeted support and development of pivotal stakeholders—those specifically responsible for the conceptualisation, design, implementation and evaluation of assessment in their clerkships. The findings do not represent other academics involved in assessing students, nor did we specifically explore how different groups of assessors (e.g., according to career stage) might behave.

Utility of this model to guide change initiatives for assessment change is a priority, which also requires an exploration of the strength of interactions between personal and contextual factors, and their relative impact on assessment action.. Whether there is utility to ascertaining whether the model can discern patterns for different demographic groups can also be explored. This could enable more effective interventions through identification of priority factors (i.e., targeting factors with the greatest relative impact on assessor behaviour) or bespoke approaches for different groups of faculty. Future research should also investigate what factors may be in play when intention succeeds to transition into action.

Moreover, the distinction between personal competencies (C1) and environmental affordances and constraints versus their overlap with other personal and contextual factors, such as perceived self-efficacy (A2), external response costs (A1b), and the physical and organisational environment (B2) respectively, need to be clarified.

Additionally, future research should elucidate the relationship between individuals’ conceptions of assessment, which were shown to influence assessment practice (Sims & Cilliers, 2023a), and the HBT factors reported here. We speculate that convenor assessment literacy could be developed through HBT’s recommended assessment-related events and interventions (B2a), and increased access to assessment-related information, products and services (B2b), which in turn could progress assessor conceptions of assessment along the continuum, from less to more sophisticated, with concurrent changes to assessment practice. It would be interesting to explore whether conceptions of assessment provide an overarching moderating framework that shapes individuals’ responses to each HBT factor, or whether they play a role in the intention to action transition.

Lastly, it may well be that the findings from this study resonate with readers from diverse contexts globally, particularly where individual programme conveners shoulder responsibility for assessment practice. However, as with all qualitative research, transferability is for the reader to infer. This study took place in diverse Southern contexts, revealing differing distal contextual influences in terms of decolonial (e.g., localised assessment practices in South Africa) and neo-colonial (e.g., internationalised or globalised assessment practices in Mexico) responses, and we trust that the detail provided about the context, the participants and the findings will enable readers to make appropriate inferences about the applicability of these findings to other contexts.

## Conclusion

Assessment done for certification purposes in medicine is of critical importance due to its significant consequences if done poorly. While, in the global North, programmatic assessment drawing on the collective competency of an assessment team is becoming more widespread, in many global South contexts, with their differing social, historical, political contexts and resource constraints, other more feasible assessment practices dominate and are likely to persist. In these settings, individuals are responsible for the design and implementation of assessment at course- and programme-levels. Interventions to enhance assessment therefore needs to address individual clerkship convenors in their contexts. Understanding how these individuals practice assessment is the first step toward enhancing assessment practices. In order to support quality assessment practices in these contexts, this study explored the high stakes, exit-level clerkship assessment practices of individual convenors responsible for assessment in varied Southern contexts. A holistic and explanatory model of assessor behaviour was developed, with implications for multi-factorial, multi-level faculty development.
